# Intensity of nursing work in public hospitals*

**DOI:** 10.1590/1518-8345.3221.3267

**Published:** 2020-05-11

**Authors:** Tatiane Araújo dos Santos, Handerson Silva Santos, Elieusa e Silva Sampaio, Cristina Maria Meira de Melo, Ednir Assis Souza, Cláudia Geovana da Silva Pires

**Affiliations:** 1 Universidade Federal da Bahia, Escola de Enfermagem, Salvador, BA, Brazil.

**Keywords:** Work, Nursing, Nurses, Nurses’ Aides, Licensed Practical Nurses, Hospitals, Public, Trabalho, Enfermagem, Enfermeiras e Enfermeiros, Auxiliares de Enfermagem, Técnicos de Enfermagem, Hospitais Públicos, Trabajo, Enfermería, Enfermeros, Auxiliares de Enfermería, Enfermeros no Diplomados, Hospitales Públicos

## Abstract

**Objective:**

to analyze the intensity of nursing work in public hospitals.

**Method:**

cross-sectional, quantitative study, carried out in 22 public hospitals. The sample was composed of 265 nurses and 810 nursing technicians and assistants. Data were collected through a questionnaire and analyzed with Exploratory Factor Analysis. The calculation of the distribution of the work intensity by category was done using a score ranging from -1 to +1 standard deviation of the data. Fisher’s exact test (0.05 <p≤0.10) was used to observe the significance between groups according to the employment bond.

**Results:**

work intensity contributed to the explanation of precarization of work, with a value of 13% for nurses and 51.2% for technicians and assistants. For the technicians and assistants, the variables with the highest factor loadings were ‘*work requires more than she can do*’ (0.6696) and ‘*takes on multiple tasks due to staff shortages*’ (0.6156). Among nurses, the highest factor loadings were observed in the variables ‘*time pressure at work* (0.6779) and ‘*Work pace*’ (0.6651).

**Conclusion:**

the variables analyzed indicate that work intensity occurs differently among nursing workers, and is revealed by the versatility, understaffing and flexibility of work.

## Resumo

**Objetivo:**

analisar a intensidade do trabalho em enfermagem nos hospitais públicos.

**Método:**

estudo transversal, quantitativo, realizado em 22 hospitais públicos. A amostra totalizou 265 enfermeiras e 810 técnicas e auxiliares em enfermagem. Os dados foram coletados por meio de questionário e analisados com a Análise Fatorial Exploratória. O cálculo da distribuição da intensidade do trabalho por categoria foi feito por escore que abarca de -1 a +1 desvio padrão dos dados e utilizou-se o teste Exato de Fisher (0,05 <p≤0,10) para observar a significância entre os grupos de acordo com o vínculo de trabalho.

**Resultados:**

a intensidade do trabalho contribuiu com 13% da precarização do trabalho para as enfermeiras e 51,2% para as técnicas e auxiliares. Para as técnicas e auxiliares as variáveis com maior carga fatorial foram *trabalho exige mais do que pode fazer* (0.6696) e *assume múltiplas atribuições devido à escassez de pessoal* (0.6156). Entre as enfermeiras as maiores cargas fatoriais foram observadas nas variáveis *pressão do tempo no trabalho* (0.6779) e *ritmo de trabalho* (0.6651).

**Conclusão:**

as variáveis analisadas indicam que a intensidade do trabalho ocorre de forma distinta entre as trabalhadoras em enfermagem, o que se revela pela polivalência, subdimensionamento e flexibilização do trabalho.

**Objetivo:**

analizar la intensidad del trabajo en enfermería en los hospitales públicos.

**Método:**

estudio transversal, cuantitativo, realizado en 22 hospitales públicos. La muestra totalizó 265 enfermeras y 810 técnicas y asistentes de enfermería. Los datos fueron recolectados a través de un cuestionario y analizados con el Análisis Factorial Exploratorio. El cálculo de la distribución de la Intensidad del trabajo, por categoría, se realizó utilizando una puntuación que varía de -1 a +1 desviación estándar de los datos, y se utilizó la prueba Exacta de Fisher (0,05 <p≤0,10) para observar la significancia entre grupos según la relación laboral.

**Resultados:**

la Intensidad del trabajo contribuyó con el 13% de la explicación *trabajo precario* para las enfermeras y el 51,2% para técnicas y asistentes. Para las técnicas y auxiliares, las variables con la carga de factor más alta fueron *trabajo requiere más de lo que puede hacer* (0,6696) y *asume múltiples asignaciones debido a la escasez de personal* (0,6156). Entre las enfermeras, las cargas de factor más altas se observaron en las variables *presión de tiempo en el trabajo* (0,6779) y *ritmo de trabajo* (0,6651).

**Conclusión:**

las variables analizadas indican que la intensidad del trabajo ocurre de manera diferente entre los trabajadores de enfermería, lo que se revela por la versatilidad, por condiciones laborales reducidas y la flexibilización del trabajo.

## Introduction

Control over work time has always been the focus of employers in capitalist society. The class that owns the means of production seeks to control this time, enforcing the idea that it is quantitative and rational. This ideological construction is deemed as universal and as the standard in modern society^(^ 1 ^)^.

To understand the use of work time in the perspective of the employer, it is necessary to address its three dimensions: duration of this time (working hours), distribution of the time and work intensity. Duration of working hours refers to the total time of work. It is the most perceptible dimension and, for this reason, it is at the center of the conflict between unions and employers. The distribution of working time is its organization in days, weeks, months, years. This distribution includes intermittent work, compensatory time, home office or remote work and all the free time used to gain qualifications for work^(^ 2 ^-^ 3 ^)^.

Work intensity is a term that still does not have a consensual definition in the field of sociology of work. In this article, the definition used was “intensity is the effort made by workers to meet the constraints of work organization during a unit of time”^(^ 2 ^)^.

Therefore, work intensity does not only mean extending working hours, but refers to the worker’s ability to produce more work on the same workday. For this reason, this intensity is difficult to be perceived, as it is necessary to investigate the constraints that lead the worker to spend more physical, psychological, mental, social, cultural and relational energy during their work^(^ 1 ^-^ 3 ^)^.

It should be noted that work intensity has an uneven impact on outsourced and permanent employees and on men and women, as the latter are subject to more intense work^(^ 3 ^-^ 5 ^)^. Considering that the professional field of nursing in Brazil is composed mainly of women^(^ 6 ^)^, the authors of the present study choose to use feminine terms to give visibility to the intensity of women’s work.

Work intensity is also revealed by fast workflows, in which the chain of activities must be done quickly to not disrupt the continuous flow of collective work. Intense work and fast workflows are increasingly common in hospitals and are related to the way work is organized^(^ 4 ^-^ 5 ^)^. The work organization model in nursing combines characteristics of toyotism and taylorism, which contributes to the intensification of work and subjects’ workers to organizational constraints.

Intense work and fast workflow are characteristics of nursing work^(^ 4 ^-^ 5 ^)^. Studies reveal that work intensity has serious consequences for the nursing staff, such as professional errors, work accidents and physical and psychological illness^(^ 7 ^-^ 10 ^)^.

Intensity is a form of precarization of labor. The enforcement of goals, extended working hours and the necessity for versatility of the worker are aspects related to intensity that hinder work relationships and work conditions. It should be mentioned that all these aspects are supported by fear-based management and moral harassment, which compels workers to submit themselves to the intensity of work^(^ 3 ^,^ 11 ^)^.

In a review of the state-of-the-art studies on work intensity on the Virtual Health Library platform, limited to publications between the years 2015 and 2018, due to the increase of publications on this topic in this period, using the boolean operator “and” and accepting only full text articles, a total of 13 articles addressing the intensity of nursing work were found. However, these articles address work intensity as one of the factors related to stress or illness of the nursing worker, without relating it to the contemporary situation of precarization of labor.

That said, the objective of this article is to analyze the intensity of nursing work in public hospitals.

## Method

This is a cross-sectional, quantitative study, carried out in 15 (fifteen) public hospitals directly administered by the Health Secretariat of the State of Bahia and 7 (seven) hospitals indirectly administered by this Office. The hospitals agreed to participate in the research and provided records with the number of nurses, nursing technicians and nursing assistants.

The software STATA version 11 was used to estimate the size of the population, considering the information provided in the records mentioned above. As the prevalence of the studied phenomenon was unknown (p=0.50), sampling error was set at 3% (d=0.03) and confidence interval at 95% (α=0,05). The total elements in each stratum corresponded to the total number of nursing workers registered in organizations with direct management (n=7,140, with 1,712 nurses, 2,597 nursing technicians and 2,831 nursing assistants) and indirect management (n=1,681, with 436 nurses, 1,160 nursing technicians and 85 assistants).

The stratified sampling with proportional allocation of workers was calculated according to the type of administration, work category and employment bond, totaling 265 nurses (n=161 permanent and n=104 outsourced) and 810 nursing technicians and assistants (n=597 permanent and n=213 outsourced).

Data was collected between March 2016 and February 2017 by the researchers and fellowship holders of the research group. The technique used for data collection was a survey conducted with a questionnaire containing 96 questions divided into seven sessions: I. Socio-demographic characteristics, II. Information on other employment bonds; III. Information on this work; IV. Information on the work process; V. Information on working conditions; VI Information on domestic activities and VII. Information on salary. The elaboration of the instrument was supported by an extensive literature review, the experience of nurses working in health organizations and instruments used in other studies.

The instrument validation process occurred from January to February 2015, with 347 workers from a hospital organization that was not included in the sample but had characteristics similar to those expected in the data collection fields. There was a need to change the wording of some questions to facilitate understanding. The collection instrument was also validated in multiple workshops, in which nursing workers and nurses with expertise on nursing work participated. There was subsequent guidance from a professional specialized in statistics.

The selection system adopted was direct approach to active workers in their respective sectors. Inclusion criteria were: nurses, nursing technicians and nursing assistants with more than 6 months of work^(^ 12 ^)^ in the health institution and being able to answer the questions, that is, understanding what is being asked, considering that this ability is not necessarily related to the level of education.

Exploratory Factor Analysis (EFA) was used for data analysis. This method allows to reduce data, looking for latent variables (factors) that have high explanatory power in relation to the object of study. The main reasons for the use of EFA are the validation of the results of an evaluation and the development of a theory, since it grasps the construct and the synthesis of its relationships, resulting in factors that can be used in other analyzes^(^ 13 ^)^.

The following steps were followed for the use of EFA: 1. Based on a study on the typologies of the precarization of labor^(^ 11 ^)^, variables were allocated to the construct ‘work intensity’, building a factorial matrix; 2. The matrix was factored according to the category of the worker and type of employment bond. The Kaiser-Meyer-Olkin (KMO) test was performed, obtaining a global score of 0.8307 for nurses and 0.8794 for nursing technicians and assistants. Cronbach’s alpha coefficient was used to validate the internal consistency of the variables; the global score of the nurses’ matrix was 0.8822, while the matrix of technicians and assistants obtained a score of 0.8839, which indicate high homogeneity of responses.

The scree test was used for the extraction of the factors. Factor rotation was resumed with a factor loading cutoff of 0.40. Subsequently, two methods of orthogonal factor rotation, with the varimax method, and one oblique rotation, with the promax method, were tested, with 4, 5 and 6 factors each, respectively. The researchers were responsible for deciding whether to use the matrix with 4, 5 or 6 factors, and if they should be dependent or independent.

After factoring, among the 96 initial variables, 11 remained in the nurses’ matrix and 13 in the nursing technicians and assistants’ matrix for the ‘work intensity’ construct.

The factor loading was calculated to assess the contribution of variables to the explanation of the construct ‘work intensity’. Eigenvalue, difference, explanation ratio, cumulative and uniqueness were calculated to verify how the work intensity contributed to the construct ‘precarization of labor’. These matrixes were discussed and evaluated in six moments with experts on nursing work, precarization of labor, epidemiology and statistics.

After the definition of the matrix of work intensity by the organization of the work process, the median, quartile 1 (Q1) and quartile 3 (Q3) were calculated to analyze the workers’ responses. A Likert scale with the alternatives 1 = never, 2 = rarely, 3 = sometimes, 4 = frequently and 5 = always was used.

In order to observe how work intensity was distributed by type of employment bond and by category of worker, a score ranging from -1 to +1 regarding the standard deviation (SD) of the data was created and the Fisher’s exact test (0.05<p≤0.10) was used to calculate the significance between the groups according to the employment bond. Thus, the category work intensity by the organization of the work process can present three scores:<=-1 SD low intensity; > -1SD and < + 1SD intermediate intensity; >+1SD high intensity.

This study followed the ethical precepts established in Resolution no. 466/12 of the National Health Council and was assessed and approved by the Research Ethics Committee of the Nursing School of the Federal University of Bahia, protocol 398.772/2013.

## Results

The study participants are mostly female (nurses: 90.1%; technicians and assistants: 86.9%), in the age group of 31 to 55 years (nurses: 76.9%; technicians and assistants: 82, 9%), with 6 to 15 years of experience in the profession (nurses: 79.8%; technicians and assistants: 48.1%) and of black ethnicity (nurses: 83.9%; technicians and assistants: 91.3%).

The proportion calculation shows that work intensity contributes with 51.2% of the explanation of the precarization of labor for nursing technicians and assistants (Eingevalue: 7.75547; Difference: 5.87457; Cumulative: 0.5198). For nurses, this proportion is 13.0% (Eingevalue: 2.06570; Difference: 0.20992; Cumulative: 0.5974).

Table 1 shows that, for nursing technicians and assistants, the variables related to the execution of care work have the highest factor loading. Some variables have a negative factor loading, which indicates a reverse impact on the factor, that is, they are present in daily work but are not considered relevant to work intensity.

**Table 1:** Factor Loading, Quartiles (Q1* and Q3^†^) and medians of the work intensity variables, nursing technicians and assistants (n=810). Bahia, Brazil, 2016-2017

Work intensity variables	Factor loading	Permanent			Outsourced		
		Q1*	Median	Q3^†^	Q1*	Median	Q3^†^
Work requires more than she can do	0.6696	2	3	4	2	3	4
Takes on multiple tasks due to staff shortages	0.6156	2	3	5	2	3	4
Cares for more patients than she is capable of	0.5844	2	3	4	2	3	5
Time pressure at work	0.5518	3	3	4	3	3	4
Work pace	0.5251	3	4	5	3	4	5
Does activities that are not her responsibility	0.4804	2	3	4	2	3	4
Does many things she doesn't agree with	0.4551	2	3	3	2	3	3
Feels pressured by her boss to complete tasks	0.4542	1	2	3	1	2	3
Does activities for which she was not trained	0.4328	1	2	3	1	2	3
Tasks are interrupted before she can complete them	0.4323	1	2	3	1	2	3
Rest breaks during the workday	-0.4510	2	3	4	2	3	4
Work allows the development of activities and the achievement of goals in a calm manner	-0.4863	2	3	4	2	3	4
Eats calmly during the workday	-0.5046	2	3	4	2	3	4

* Q1= Quartile 1;

† Q3 = Quartile 3

The Quartiles for nursing technicians and assistants showed a difference in two variables when comparing the groups of permanent and outsourced workers. In the variable ‘takes on multiple tasks due to staff shortages’, Q3 is higher for permanent workers (5=always) than for outsourced workers (4=frequently). In the variable ‘cares for more patients than she is capable of’, Q3 is higher (5=always) for the group of outsourced workers than for permanent workers (4=frequently) (Table 1).

For nurses, the highest factor loading is in the variable related to time pressure to perform tasks, that is, nurses feel that they have less time to perform an increasing number of tasks (Table 2).

**Table 2:** Factor Loading, Quartiles (Q1* and Q3^†^) and medians of the work intensity variables, nurses (n=265). Bahia, Brazil, 2016-2017

Work intensity variables	Factor Loading	Permanent			Outsourced		
		Q1*	Median	Q3^†^	Q1*	Median	Q3^†^
Time pressure at work	0.6779	3	3	4	3	3	4
Work pace	0.6651	4	5	5	4	4	5
Work requires more than she can do	0.6052	2	3	4	3	3	4
Cares for more patients than she is capable of	0.5700	3	4	5	3	4	5
Takes on multiple tasks due to staff shortages	0.5494	2	4	4	3	4	4
Supervises more workers than she is capable	0.4307	1	3	4	2	2	4
Tasks are interrupted before she can complete them	0.4140	2	3	4	2	3	3
Repetitive gestures	0.4039	3	4	5	3	4	5
Rest breaks during the workday	-0.4589	1	2	3	1	3	3
Work allows the development of activities and the achievement of goals in a calm manner	-0.4946	2	3	3	2	3	3
Eats calmly during the workday	-0.5517	2	3	4	2	3	4

* Q1= Quartile 1;

† Q3 = Quartile 3

Differences in responses between the groups of permanent and outsourced workers are expressed in six variables. In the variable ‘work pace’, the median of the responses of permanent workers was 5 (always), while for outsourced workers it was 4 (frequently).

In the variable ‘work requires more than she can do’, the Q1 for permanent nurses was 2 (rarely), while for outsourced nurses it was 3 (sometimes). For 25% of permanent workers, the variable ‘takes on multiple tasks due to staff shortages’ is a situation that occurs rarely (2). For outsourced nurses, this condition occurs sometimes (Q1=3).

‘Supervision of more workers than they are capable’ never occurs for 25% of permanent workers (Q1=1). For outsourced workers, this situation rarely occurs (Q1=2). For 75% (Q3) of the permanent workers, ‘tasks are often interrupted before they can complete them’; for outsourced workers, this happens sometimes (Q3=3). Permanent nurses rarely (Median = 2) ‘pause to rest during work’, while outsourced nurses do it sometimes (3).

Regarding the scores of precarization variable by intensity by the organization of the work process (Table 3), there is no statistically significant difference between the groups of permanent and outsourced workers for nursing technicians and assistants (p=0.431). However, when analyzing proportions, it is observed that outsourced workers are more frequently in the intermediate (62.0%) and high (21.6%) strata than permanent nursing technicians and assistants (intermediate=60.5% and high=19.3%).

**Table 3:** Work intensity score for nursing technicians and assistants, according to type of employment bond. Bahia, Brazil, 2016-2017

Work intensity score	Permanent		Outsourced		p-value
	N	%	n	%	
<=-1SD* low	121	20.3	35	16.4	0.431
>-1SD* and <=+1SD* intermediate	361	60.5	132	62.0	
>+1SD* high	115	19.3	46	21.6	
Total	597	100.0	213	100.0	

* SD = Standard deviation

There is also no statistically significant difference between the proportions of the groups of permanent and outsourced nurses (p = 0.449) for the work intensity score (Table 4). However, there’s a higher proportion (21.7%) of permanent nurses in the high stratum of work intensity than of outsourced nurses (15.4%).

**Table 4:** Work intensity score for nurses, according to type of employment bond. Bahia, Brazil, 2016-2017

Work intensity score	Permanent		Outsourced		p-value
	N	%	n	%	
<=-1SD* low	28	17.4	19	18.3	0.449
>-1SD* and <=+1SD* intermediate	98	60.9	69	66.3	
>+1SD* high	35	21.7	16	15.4	
Total	161	100.0	104	100.0	

* SD = Standard deviation

## Discussion

According to the definition of intensity^(^ 2 ^)^ used in this article, workers use their workforce to tackle the deficits and constraints of the health organization and enable the continuity of care, which is an evidence of the intensity of their work.

The work intensity matrixes reveal the distinct positions of workers within the work process. For nursing technicians and assistants, the variables related to direct assistance to the user obtained the highest factor loadings. This means that these workers are more prone to illness and care errors due to their high workload^(^ 14 ^-^ 15 ^)^.

For nurses, the variables reflected the position in management and care that these professionals occupy in the nursing work process. For these professionals, work intensity has an impact on decision making, compromising the management part of their work. In addition, the performance of care and management activities at the same time makes nurses more prone to perceive the intensity of their work^(^ 16 ^-^ 17 ^)^.

Work intensity is one of the aspects of precarization of labor, which has the objective of dominating the worker through fear^(^ 11 ^)^, based on the new demands of the current model of production - toyotism - which requires versatility, multifunctionality and flexibility. In the current context, being flexible and multifunctional means accepting changes in the short term, meeting the demands of the employer with agility, taking risks, negotiating at work without being based on rules and regulations, being available at all times for work demands or training using free time^(^ 1 ^,^ 3 ^)^. As a result, work intensity adds to the vulnerability of new forms of hiring, unhealthy working conditions and loss of labor rights, which increases and reinforces the precarization of labor.

Studies in the field of sociology reveal that precarization of labor does not occur evenly among workers. Women, black men and women, young adults and outsourced workers are more vulnerable to this phenomenon and, therefore, are also more subject to work intensity^(^ 11 ^,^ 18 ^)^.

The variables associated with work intensity in this study demonstrate that versatility and flexibility are current characteristics of nursing work in public hospitals in Bahia/Brazil. However, studies carried out in other countries^(^ 19 ^-^ 23 ^)^ found that work intensity is a global phenomenon, and work versatility is its main characteristic.

Among the factors that intensify nursing work, understaffing stands out. This has become a common *modus operandi* in all institutions^(^ 4 ^).^. This reveals the adoption of flexibility in nursing work, that is, there is an increase in the number of patients and in the degree of complexity and, consequently, the care procedures required are always performed by less staff than necessary^(^ 4 ^)^.

Understaffing, associated with the variables related to versatility, puts at risk not only the health of the worker, but the patients themselves. The analysis of the ethical legal cases filed in the Regional Nursing Council of Bahia showed that, all the times that a worker made a mistake, the work intensity caused by understaffing was a causal factor^(^ 22 ^)^. An American study on the impact of intensity and understaffing on patient safety revealed that, for every additional patient that was admitted to the investigated Intensive Care Unit (ICU), increasing the nurse’s workload, the odds of patient mortality increased by 7%^(^ 23 ^)^.

In this context of understaffing, in which the worker needs to take on multiple functions, other characteristics of toyotism appear. An intense work pace and time pressure can increase productivity at work, which has always been the objective in the capitalist mode of production^(^ 1 ^-^ 3 ^)^.

Permanent workers do not get profit from selling their workforce. Therefore, for them, this increase in productivity means that nurses, technicians and assistants have high work demands, even though they do not always have the necessary conditions for this. Therefore, they partially meet the constraints of the State in the provision of hospital services^(^ 11 ^,^ 18 ^)^.

For outsourced workers, selling their workforce creates surplus value for the outsourcing companies. Thus, the increase in productivity and the understaffing allow companies to increase their profit^(^ 11 ^,^ 18 ^)^, given that they do not hire the necessary quantity of nurses, technicians and assistants and can extract more work from their staff, reaching the goals agreed with the State - their contractor.

However, a study on the outsourcing of nursing services in an Iranian public hospital revealed its lack of efficiency, mainly because after outsourcing the costs per bed increased, productivity decreased and a positive variation of 1% was found in customer satisfaction^(^ 24 ^)^.

All variables analyzed can lead to physical and emotional exhaustion of nurses, technicians and assistants, and can also explain the desire to leave the profession expressed by these workers^(^ 25 ^)^, work-related illness^(^ 26 ^-^ 27 ^)^ and/or professional errors^(^ 22 ^,^ 28 ^)^.

The similarity of the answers in the quartiles and medians and the lack of statistical difference in work Intensity score between permanent and outsourced nurses, technicians and assistants demonstrates that, regardless of the type of employment, work intensity is a fact for all these workers. Some studies in the area of sociology of work^(^ 29 ^-^ 31 ^)^ reveal that outsourced workers are more vulnerable to work intensity than other workers; however, this was not observed in this research.

The limitations of this study were the long data collection period, due to the conditions of the research field, and the fact that it was carried out only in public hospitals.

An advancement of scientific knowledge in the field of nursing that stands out in this study is the approach to work intensity as a central element for the precarization of work, revealing that this phenomenon is uneven for the three categories of workers. Therefore, specific studies on work and intensity according to the professional category should be performed.

## Conclusion

For nursing workers in public hospitals in Bahia, work intensity was revealed by variables related to understaffing and versatility and flexibility in the execution of tasks and activities.

The results of the factor loadings demonstrate that work intensity variables vary between the professional categories and are related to the work performed. Therefore, for nursing technicians and assistants, the variables that most intensify work are those related to direct assistance; for nurses, the variables associated with the management of the work process are the most relevant.

The lack of statistically significant difference between the groups of outsourced and permanent workers in the precarization of labor by intensity score reveals that nurses, technicians and nursing assistants are subject to this condition in the hospitals of the Unified Health System.

Since intensity of work is one of the aspects of precarization, it will become more evident as work becomes more precarious, which for nursing work means long hours and low wages, but also understaffing, versatility and flexibility.
